# Olfactory fMRI Activation Pattern Across Different Concentrations Changes in Alzheimer’s Disease

**DOI:** 10.3389/fnins.2019.00786

**Published:** 2019-07-30

**Authors:** Hui Zhang, Dongxu Ji, Jianzhong Yin, Zhiyun Wang, Yuying Zhou, Hongyan Ni, Yawu Liu

**Affiliations:** ^1^Department of Radiology, Tianjin First Central Hospital, Tianjin, China; ^2^Department of Radiology, First Affiliated Hospital of Tianjin University of Traditional Chinese Medicine, Tianjin, China; ^3^Department of Neurology, Tianjin First Central Hospital, Tianjin, China; ^4^Department of Neurology, Tianjin Huanhu Hospital, Tianjin, China; ^5^Departments of Clinical Radiology and Neurology, Kuopio University Hospital, University of Eastern Finland, Kuopio, Finland

**Keywords:** Alzheimer’s disease, mild cognitive impairment, olfactory fMRI, primary olfactory cortex, olfactory habituation

## Abstract

The olfactory involvement is an early feature of Alzheimer’s disease (AD). Olfactory functional MRI (fMRI) is an objective method to evaluate the olfactory function, but might be affected by the individual variation and the magnetic susceptibility artifact of basis cranii. To improve the reliability of olfactory fMRI, we explored the response of primary olfactory cortex (POC) across three different concentrations of odors. Fourty-four normal controls, 46 subjects with mild cognitive impairment (MCI), and 44 patients with AD underwent olfactory fMRI using lavender stimuli of three different (0.10, 0.33, and 1.00%) concentrations during one fMRI sequence with a 3.0T MRI scanner. The numbers of activated voxels in the POC, especially the activation changes during different concentrations were, analyzed. The POC activation pattern of controls showed olfactory adaptation at the higher concentration, whereas the AD patients showed not only increased olfactory threshold but also a lack of olfactory habituation. Five types of activation patterns across different concentrations were summarized to evaluate the olfactory function. The results showed that the activation pattern effectively found 40/44 (90.9%) of the ADs with impaired habituation, whereas 31/44 (70.5%) of the normal controls showed normal olfactory habituation. In MCIs, 29/46 (63.0%) of subjects showed impaired habituation. This finding indicates that the POC activation pattern of olfactory fMRI across different concentrations is useful in evaluating the olfactory function, which is important in the detection of early AD among MCI cases.

## Introduction

Alzheimer’s disease (AD) is the most common form of dementia affecting millions of people worldwide. In its early stage, it involves not only the entorhinal cortex but also the anterior olfactory nucleus, piriform cortex, and amygdala, then spreads to the higher order olfactory projections in the orbitofrontal cortex, insula and hippocampus (Attems et al., 2007; Wilson et al., 2007). In this stage, the patients may present olfactory disorder, but the clinical symptoms can hardly be detected (Hori et al., 2015; Velayudhan, 2015; Velayudhan et al., 2015; Vyhnalek et al., 2015). When the disease progresses, the pathological changes evolve to higher order olfactory central structures in the hippocampus and insula, then the patients can present cognitive impairment and progress to dementia in the late stage (Kerchner et al., 2012; Padurariu et al., 2012).

The University of Pennsylvania Smell Test (UPSIT) (Doty et al., 1984) is one of the most widely used clinical odor tests in identifying olfactory dysfunction. Evidence has shown that the dysfunctions of olfactory identification, discrimination, and olfactory memory exhibit before memory loss (Huttenbrink et al., 2013; Seligman et al., 2013). Among elderly persons with intact cognition, difficulty in odor identification predicts the development of mild cognitive impairment (MCI) (Seligman et al., 2013). In MCI subjects, olfactory identification deficits, particularly with a lack of awareness of olfactory deficits, is considered as an early diagnostic marker for AD (Devanand et al., 2000).

Functional MRI (fMRI) has emerged as a neuroimaging method that allows non-invasive monitoring of brain function with combined high spatial and temporal resolutions never achieved before by other imaging modalities (Cerf-Ducastel and Murphy, 2003; Wang et al., 2005). Olfactory fMRI is an objective method to detect olfactory dysfunction. Several olfactory fMRI studies have shown decreased odorant-induced activation of central olfactory structures in the healthy elderly (Suzuki et al., 2001), and decreased activated in the Primary Olfactory Cortex (POC) of early AD patients compared to healthy aged controls (Wang et al., 2010). The POC is the cortex that accepts all the olfactory afferent nerves. However, because the POC is close to the basis cranii, it is vulnerable to the magnetic susceptibility artifact which might decrease the reliability of olfactory fMRI. Moreover, it is not possible to use an activation threshold in a clinical setting due to large differences in individual olfactory fMRI activation. Consequently, it is difficult to use olfactory fMRI as a diagnostic tool in a single subject (Morrot et al., 2013). In the present study, to overcome the abovementioned obstacles, we applied three different concentration odor stimulants in one sequence scan to detect the olfactory activation. As the data during different concentrations was in the same sequence scan, the influences, including the individual olfactory difference and the artifact of basis cranii, should be same. Thus, the activation across different concentration should be more reliable to compare with each other. We hypothesized that using dynamic olfactory activation patterns could detect olfactory dysfunction without using a predefined threshold.

The aim of this study was to improve the reliability of olfactory fMRI by the evaluation of the response of POC across three different concentrations of odors.

## Materials and Methods

### Participants

The AD patients and the MCI subjects were recruited from the Neurology Clinic of local hospital and the healthy controls were recruited through community advertising. All the participants underwent the mini-mental state examination (MMSE) (Folstein et al., 1975), the Montreal Cognitive Assessment (MOCA) (Nasreddine et al., 2005), and the Clinical Dementia Rating (CDR) (Hughes et al., 1982), in addition, the associated medical history were also acquired by a neurologist with over 15 years of experience. All the AD patients met the National Institute of Neurological and Communicative Disease and Stroke and AD (NINCDS-ADRDA) criteria for a diagnosis of probable AD (McKhann et al., 1984). MCI subjects were amnestic MCI, and fulfilled the diagnostic criteria for MCI (Petersen et al., 2001a), (1) memory complaint by patient, family, or physician; (2) normal activities of daily living; (3) Mini Mental State Examination score (MMSE) range between 24 and 30; (4) Geriatric Depression Scale score less than or equal to 5; (5) Clinical Dementia Rating Scale (CDR) score of 0.5; and (6) absence of dementia according to the NINCDS-ADRDA criteria for AD. Regarding the exclusion criteria, all the participants underwent screening before admission into the study to rule out the conditions affecting olfactory function (history of head trauma, nasal diseases, respiratory infection, metabolic disorders, and serious smoking), neurological or psychiatric conditions other than AD that might adversely influence the study findings. The Hachinski ischemic Scale (HIS) (Hachinski et al., 1975) and Hamilton depression scale (HAMD) (Hamilton, 1960) were used to exclude vascular dementia and depression.

We recruited 44 AD patients (mean age 66.91 ± 8.19 years, 16 males, 28 females), 46 MCI (mean age 64.35 ± 7.14 years, 18 males, 28 females), and 44 healthy controls (mean age 63.57 ± 6.45 years, 18 males, 26 females). All the participants provided written informed consent prior to participation. This study got approval of the research ethics committee of Tianjin First Central Hospital and all methods were performed in accordance with the relevant guidelines and regulations.

### Imaging Scan

All MRI data were obtained using a 3T Siemens MRI system (Magnetom Trio Tim, Siemens Healthcare, Erlangen, Germany) and a 32-channel head coil. The subjects were positioned in a supine position. Fixing device and foam padding were used to minimize subject’s head movements. Anatomical images were acquired with a 3D MPRAGE sequence with repetition time/echo time/flip angle (TR/TE/FA) = 1900/2.5 ms/30°, field of view (FOV) = 250 × 250 mm, acquisition matrix = 256 × 256, 176 slices, thickness = 1 mm with no gap, number of average = 2. Then fMRI images covering the entire brain were acquired using echo-planar imaging with a SENSE Factor = 2, TR/TE/FA = 3000/35 ms/90°, FOV = 220 × 220 mm, acquisition matrix = 64 × 64, 25 axial slices, slice thickness = 4 mm with 0.8 mm gap and acquired for 250 frames. In addition, a fast spin-echo T2WI was also scanned to rule out other brain and sinus diseases.

### Olfactory fMRI Design

**FIGURE 1:**
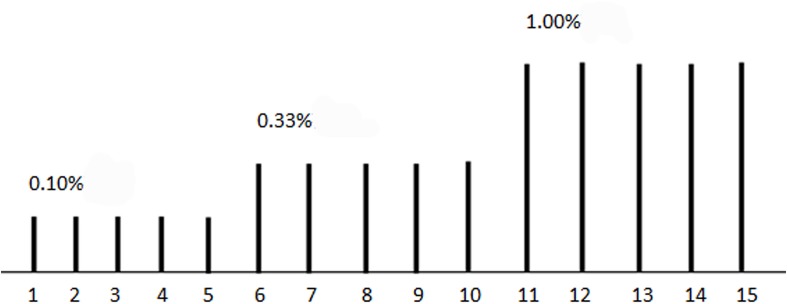
The illustration of olfactory stimuli presenting. Each stimuli lasted for 6 s, followed by a 42-s of clean odorless air, a total of 15 stimulus. 0.10, 0.33, and 1.00% lavender oil were used in the first five, second five, and third five stimulus, respectively.

We used lavender oil of three concentrations (0.10, 0.33, and 1.00%) as an olfactory stimulant (Figure 1). Lavender oil is thought to be an effective olfactory stimulant with very minimal or no propensity to stimulate the trigeminal system and has been used in olfactory fMRI study. Olfactory stimuli were delivered through a nasal cannula with a custom-built olfactorymeter, about 1 cm away from the participant’s nose with an air flow at 8 L/min. Each concentration of the stimuli was repeated for five times. Each odor stimulant was applied for 6 s and followed by a 42-s of clean odorless air, beginning with the lowest concentration. Before MR scanning, we explained the progression of the examination, and instructed the subjects to keep head and body motionless throughout examination, receiving the stimulus odor without sniffing.

### Data Processing

We used Matlab 7.11.0 and SPM8 (Statistical Parametric Mapping 8) software for data processing. The images were realigned to remove minor head movement (<2 mm). The T1-weighted high-resolution anatomical images were co-registered and spatially normalized to the Montreal Neurological Institute (MNI) brain template (Collins et al., 1998) with a spatial resolution of 1 mm × 1 mm × 1 mm. The time-course images were normalized using the same normalization parameters with a spatial resolution of 3 mm × 3 mm × 3 mm, then were smoothed with an 8 mm × 8 mm × 8 mm Gaussian smoothing kernel. The statistical parametric map was generated using preprocessed images of each subject for the whole paradigm and each concentration, according to the activation model of event-related design parameters (*P* < 0.001 and no cluster threshold).

**FIGURE 2:**
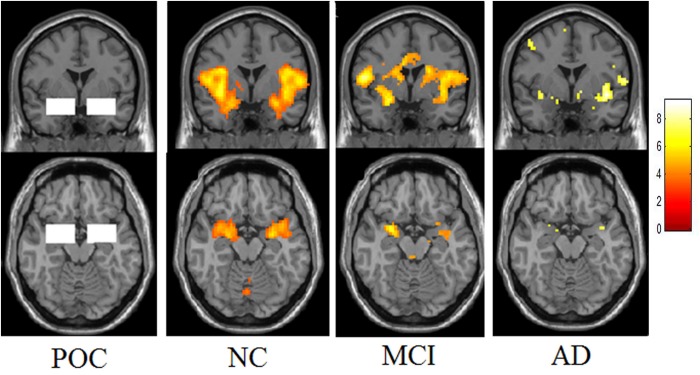
The region of POC and activation maps of the three groups. This picture demonstrates the ROI of the POC region. Presenting the lavender stimuli of all three different concentrations, the NC showed obvious activation in the bilateral POC. The MCI showed decreased activation and the patients with Alzheimer’s disease showed the lowest activation.

In addition to the whole brain activation analysis, a region of interest (ROI) analysis was performed to measure the activated voxel numbers in the bilateral POC of these three groups (Figure 2). Two 34 mm × 22 mm × 20 mm ROIs were selected at the left and right POC, the centers were −24, 0, −17 and 24, 0, −17 in the MNI space. The ROI of POC was co-determined by neurologists, radiologists and neuropathologists (Wang et al., 2010). It begins from the posterior orbitofrontal cortex and continues caudally through the most rostral-medial aspects of the temporal lobe to the level posterior to the optic chiasm. The POC structure includes the piriform cortex and closely associated areas of the anterior olfactory nucleus, the anterior perforated substance, and the olfactory tubercle, as well as the periamygdaloid cortex and the amygdalae. The activated voxel numbers of POC of the whole paradigm and each concentration were all evaluated.

### Statistical Analysis

Differences in demographic and disease related measures were assessed with one-way analyses of variance (ANOVA) followed by Bonferroni *post hoc* tests among the three groups. We used χ^2^-tests to assess the differences in gender and POC activation patterns among the three groups. The correction of *P*-value for multiple comparisons was multiplied by the number of comparisons. *P* < 0.05 was considered as statistical significance in all statistical tests.

To compare the diagnostic efficiency of the activation pattern and activated voxel numbers of POC to distinguish the AD and NC groups, the sensitivity, specificity, positive and negative predictive values, the Youden index was calculated and ROC curves was analyzed.

## Results

### Demographics of the Participants

The AD, MCI, and NC groups did not differ in age, gender, and education years. But the MMSE and MOCA scores were significantly different from each other. The MMSE and MOCA scores of AD patients (19.23 ± 5.81 and 13.14 ± 5.01) (mean ± SD, same below) were significantly lower than that of MCI (27.22 ± 1.37 and 22.43 ± 2.17), and lower than that of the NC group (28.89 ± 1.08 and 27.05 ± 1.03). A summary of the demographic and cognitive scores was presented in Table 1.

**TABLE 1:** Subjects characteristics (mean ± SD).

**Group**	**n**	**Gender (M/F)**	**Age**	**Education**	**MoCA**	**MMSE**
NC	44	18/26	63.57 ± 6.45	10.27 ± 3.30	27.05 ± 1.03^*^^#^	28.89 ± 1.08^*^^#^
MCI	46	18/28	64.35 ± 7.14	9.46 ± 4.31	22.43 ± 2.17^#∧^	27.22 ± 1.37^#∧^
AD	44	16/28	66.91 ± 8.19	9.95 ± 3.78	13.14 ± 5.01^*^^∧^	19.23 ± 5.81^*^^∧^

*F*-value		0.026^Δ^	2.535	0.522	217.07	97.48
*P*-value		0.975	0.083	0.595	< 0.001	< 0.001

^Δ^Represents the *χ*^2^ value. The MCI, AD, and NC did not different with respect to age, gender, and education years. ^*^Represents significantly difference between AD and NC groups of MoCA and MMSE; ^#^Represents significantly difference between MCI and NC groups of MoCA and MMSE; ^∧^Represents significantly difference between MCI and AD groups of MoCA and MMSE.

### Whole Brain Analysis

There was a decrease tendency in brain activation across NC, MCI, and AD groups. The AD groups showed low activation in multiple brain areas compared with NC groups, mainly included bilateral insular lobe, temporal lobe, limbic lobe, parietal lobe, and left cerebellum (Supplement 1).

### POC Activation in ROI Analysis

The total activated voxel numbers in the POC of overall and each concentration in three groups are summarized in the Table 2. The total activated voxel numbers within the ROIs of POC of AD patients (37.91 ± 63.48) and MCI subjects (67.24 ± 91.23) were significantly lower than that of normal controls (117.70 ± 150.00) (*P* < 0.001 and *P* = 0.028), but there were no significant differences between MCI and AD patients (*P* = 0.198). The activation maps at level of the POC in the three groups are displayed in Figure 2.

**TABLE 2:** The activated voxel numbers in the POC of the three groups.

		**The activated voxels of the POC (mean ± SD)**			
**Group**	**N**	**All**	**0.10%**	**0.33%**	**1.00%**
NC	44	117.70 ± 150.00^*^	70.64 ± 97.82^*^	101.09 ± 151.61^*^	37.66 ± 104.72
MCI	46	67.24 ± 91.23	34.74 ± 68.59	54.50 ± 118.34	69.35 ± 125.08
AD	44	37.91 ± 63.48^*^	13.86 ± 34.59^*^	23.57 ± 45.04^*^	38.07 ± 61.28

*F*		6.201	7.050	5.148	1.467
*P*^#^		0.012	0.004	0.028	0.936

^*^Represents significantly difference between AD and NC groups; *^#^*Corrected *P*-value for multiple comparisons.

**FIGURE 3:**
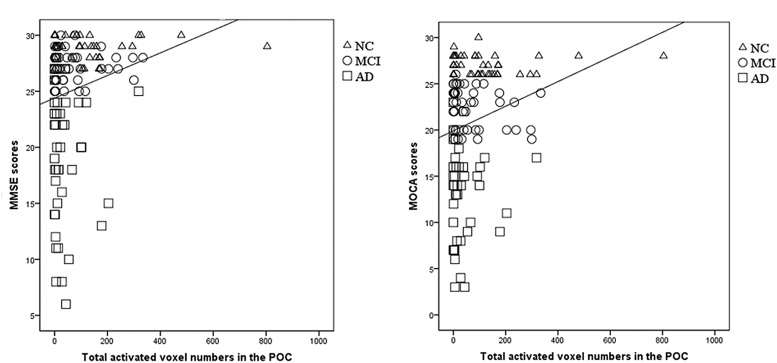
The correlation between the POC activation and MMSE/MOCA scores. **(A)** There was a significantly correlation between the total activated voxel numbers in the POC and the MMSE scores (*r* = 0.207, *P* = 0.016). **(B)** There was a significantly correlation between the total activated voxel numbers in the POC and the MOCA scores (*r* = 0.226, *P* = 0.009). From this figures, the overlapping distribution of POC activation for NC, MCI, and AD subjects could also be found, which implied it’s impossible to setup a threshold to separate these groups.

The total activated voxel numbers within the POC showed significant correlation with MMSE and MOCA scores (*r* = 0.207, *P* = 0.016 and *r* = 0.226, *P* = 0.009, respectively; Figure 3).

### POC Activation Patterns Across Different Concentration: Group Level

**FIGURE 4:**
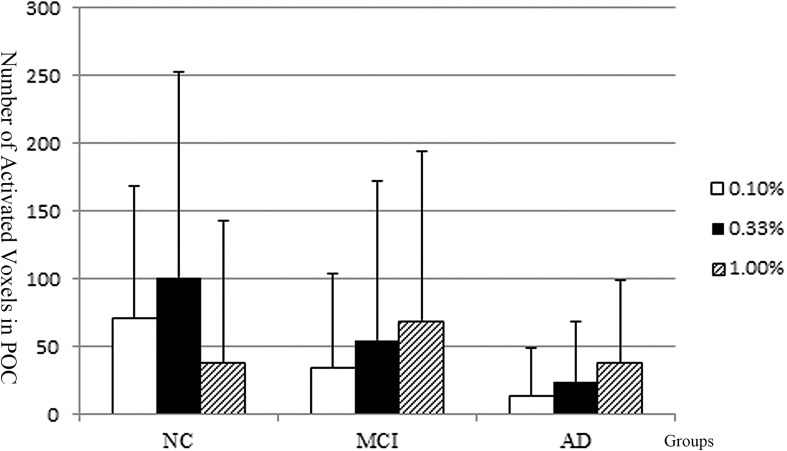
The POC activation pattern across different concentration of the NC, MCI, and AD groups. The NC implied the normal olfactory habituation to the higher concentrations. The Alzheimer’s disease patients implied lacking of olfactory habituation. And the activation pattern of the MCI group is similar to the AD.

The activated voxel numbers within the POC across the low, median, and high concentrations of the three groups are presented in Figure 4. The activations across different concentrations of AD and NC groups showed distinct patterns.

In the NC group, the number of activated voxels within the POC was low during low concentration (0.1%); then was increased during the median concentration (0.33%); but during the highest concentration (1.0%), the number of activated voxels was decreased, which implies the POC areas had normal function of olfactory habituation.

In the AD patients, the number of activated voxels within the POC was the lowest at the low odorant concentration (0.1%); then was slightly increased as the odorant concentration increased to the median (0.33%); but at the high odorant concentration (1.00%), the number of activated voxels was continuously increased, which implies the POC areas not only increased the sensitivity threshold of odors, but also had dysfunction of olfactory habituation. The activation pattern across different concentrations was different in the AD from the NC groups.

In the MCI group, the overall activation pattern was similar to that of the AD group.

### POC Activation Patterns Across Different Concentration: Individual Level

**FIGURE 5:**
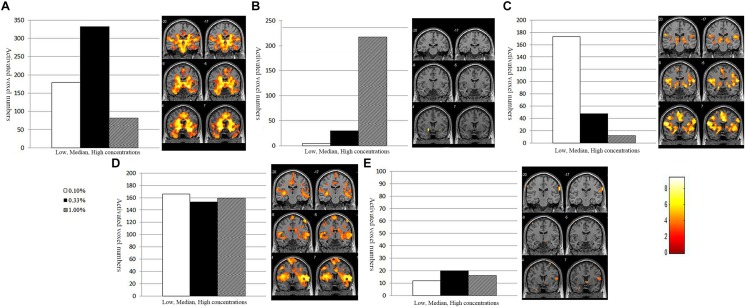
Different types of activation pattern and POC activation map. **(A)** Ascending-descending type. Bilateral POC activation was low at the low beginning, increased at the median, but decreased at the last high concentration. **(B)** Gradually ascending type. The POC activation showed a continuously increasing tendency as the concentration increased. **(C)** Gradually descending type. Bilateral POC activation was gradually decreased with the increase of odor concentration. **(D)** High plateau type. Bilateral POC activation remained at high level at all three different concentrations. **(E)** Low plateau type. Bilateral POC activation is little at three different concentrations. The **A, C, D** showed olfactory habituation and normal activation on the activation map, whereas the **B, E** implied an impaired olfactory function.

At an individual level, the POC activations in the NC, MCI, and AD presented five types: ascending-descending type, gradually descending type, high plateau type, gradually ascending type and low plateau type (Table 3 and Figure 5). A number of 15% or more of the activation changes, compared to the former concentration, would be thought of as a decrease or increase of the concentration.

**TABLE 3:** Different types of POC activation pattern of the three groups.

		**Normal habituation**				**Impaired habituation**				
**Group**	**N**	**Type A**	**Type C**	**Type D**	**Total**	**Type B**	**Type E**	**Total**	**χ2**	***P***
NC	44	15	13	3	31	3	10	13	40.25	< 0.001
MCI	46	8	9	0	17	15	14	29		
AD	44	2	2	0	4	16	24	40		

*It showed there were significantly statistical differences in the activation pattern of NC and MCI, NC, and ADMCI and AD (*P =* 0.002; *P* < 0.001).*

*A*. *Ascending-descending type* (Figure 5A) showed that POC activation was low during the beginning low concentration; then was increased during the median concentration; but, at the highest concentration (1.0%), the number of activated voxels was decreased. We could observe that the bilateral POC area activated significantly and olfactory habituation occurred at the highest concentration. Therefore, the subjects who showed ascending-descending type activation pattern had normal olfactory function.
*B*. *Gradually ascending type* (Figure 5B) showed that POC activation was the lowest at the low odorant concentration (0.1%); then slightly increased as the odorant concentration increased to the median (0.33%); but at the high odorant concentration (1.00%), the POC activation showed a continuously increasing tendency without habituation. The gradually ascending type activation pattern might imply both increased odor threshold and impaired habituation, which are the two manifestations of injured olfactory functions.
*C*. *Gradually descending type* (Figure 5C) showed that POC activation was gradually decreased with increasing concentration of olfactory stimuli. The olfactory habituation appeared at the median concentration (0.33%), which implied that the subjects who showed this type might also have a normal olfactory function.
*D*. *High plateau type* (Figure 5D) showed that POC activation was kept at a relatively high level during all of three different concentrations. The activation should increase during concentration raise, but the habituations indeed appeared. The effect of higher concentration and habituation were just almost offset or balanced in these subjects. So, the high plateau type indicates another normal expression of olfactory function.
*E*. *Low plateau type* (Figure 5E) showed the POC activation remained low level during all of three different concentrations. The three concentrations did not show significant activation, indicating this subject’s olfactory function was severely impaired.

The subjects who showed type A, C, or D (ascending-descending type, gradually descending type and high plateau type) activation pattern should have normal olfactory function, but subjects who showed type B or E (gradually ascending type and low plateau type) should have impaired olfactory function.

### Activation Pattern Analysis in Different Subjects

Throughout the olfactory activation patterns of each subject in different groups, 34.1% (15/44) of NC showed ascending-descending type (Type A), followed by the gradually descending type (Type C) (22.7%, 10/44), and 70.5% (31/44) showed normal POC activated pattern (Type A, C, or D); whereas in the AD subjects, 90.9% (40/44) of the subjects showed impaired POC activated pattern (Type B or E).

Despite that the overall activation pattern of the MCI group is similar to NCs, at the individual level only 37.0% (17/46) showed normal olfactory activation pattern (type A, C, or D), which was much lower than NCs. Whereas, 63.0% (29/46) showed type B or E pattern in the POC, which implied most MCI subjects might have olfactory dysfunction and should be follow up closely. We summarized these different patterns of subjects in the Table 3. It showed significant differences among these groups of different types (chi-square test, *P* < 0.001).

### Diagnostic Efficiency of the Activation Pattern vs. Activated Voxel Numbers of POC

The diagnostic ability of the activation pattern and activated voxel numbers of POC to distinguish the AD and NC groups were compared. In the activated voxel numbers analysis, the data of the whole paradigm (all concentrations) was used. The best estimated cut-off value for the activated voxel numbers of POC was 68, which was used to distinguish the AD and NC groups. Whereas in the activation pattern analysis, the type A, C, or D was thought as a normal olfactory function and Type B or E with no habituation effect was thought as an impaired olfactory function.

**FIGURE 6:**
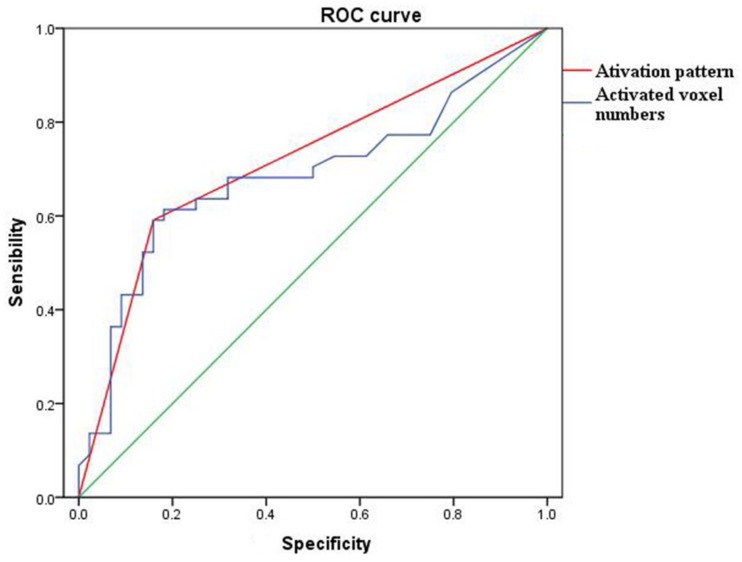
The ROC curve of activation pattern (red) and activated voxel numbers (blue) of POC to differentiate the AD cases and NC subjects. The areas under curve of activation pattern and activated voxel numbers were 0.685 and 0.716, respectively.

The sensitivity, specificity, positive and negative predictive values of the activated voxel numbers of POC were 67.27, 78.79, 84.09, and 59.09%, whereas the activation patterns were 75.47, 88.57, 90.91, and 70.45%, respectively. The Youden index of activated voxel numbers and the activation pattern were 0.46 and 0.64 (Table 4). The ROC curves also showed that the diagnostic efficiency of the activation pattern was better than the activated voxel numbers of POC (AUC 0.716 and 0.685, respectively) (Figure 6).

**TABLE 4:** The diagnostic efficiency of activation pattern vs activated voxel numbers of POC.

	**Groups**	**Normal olfactory**	**Olfactory dysfunction**	**Sensibility**	**Specificity**	**Positive predictive value**	**Negative predictive value**	**Youden index**
Activated voxel numbers	NC	26/44	18/44	67.27%	78.79%	84.09%	59.09%	0.46
	AD	7/44	37/44					
Activation pattern	NC	31/44	13/44	75.47%	88.57%	90.91%	70.45%	0.64
	AC	4/44	40/44					

*The cut off value of activated voxels to classify the normal and olfactory dysfunction was 68.*

## Discussion

Our results demonstrated that normal controls and AD patients presented different activation patterns in the POC across different concentrations, suggesting that applying different concentration stimuli in one fMRI sequence is a good way to evaluate the olfactory function, especially in individual cases, which might potentially be useful in early diagnosis of MCI and early AD cases at clinical setting.

The habituation is a characteristic of olfactory which is different from other sensory systems. The habituation generally would appear about half minute after continuous exposure to an odor in healthy subjects. But in our fMRI paradigm which used multiple short odor stimuli with increased concentrations, the habituation would appear at the third highest concentration in most normal controls. For the reason that the habituation could decrease the activation of POC, some researchers tried to avoid this effect to get more activation (Karunanayaka et al., 2014). On the other hand, the habituation effect could help us to evaluate the olfactory function which is important in the diagnosis of early AD. Despite the susceptibility artifact, there were still a correlation of the POC activation and the odor stimuli (Lu et al., 2018). The olfactory dysfunction could manifest as an elevated olfactory threshold and an impaired habituation. The elevated olfactory threshold should appear as the low activation number at the POC. However, the activation number might also be affected by the individual difference. We used three concentrations for detail evaluated the olfactory response and the habituation effect. A higher odor concentration should have a higher POC activation response. The habituation would manifest as a decrease of activation at the later higher concentration which should have a higher activation. And our definition of normal “habituation” is the activation of higher concentration that did not increase by 15% or more than the former lower concentration. A loss of habituation at the higher concentration implied an impaired olfactory function.

The POC plays a key role during the olfactory function (Vasavada et al., 2015). Olfactory afferent nerve fibers through the olfactory bulb reach the POC, and exchange of neurons, then send out the projection fibers to the higher order olfactory central structures in the orbitofrontal cortex, insula and hippocampus (Christen-Zaech et al., 2003; Wilson et al., 2007). The POC is the cortex that accepts all the olfactory afferent nerves, it includes the anterior olfactory nucleus, piriform cortex, periamygdaloid cortex, amygdala, and entorhinal cortex (Gottfried et al., 2006; Howard et al., 2009).

In our study, most of NCs and some MCI subjects showed olfactory habituation of POC. Other olfactory fMRI studies have also shown that the POC can exhibit rapid olfactory habituation when stimulated by the same odor continuously in normal people (Sobel et al., 2000; Poellinger et al., 2001). In the present study, the number of activated voxels within POC decreased as the odorant concentration increased in healthy people, which is in line with the findings by Wang et al., that normal POC presents olfactory habituation (Wang et al., 2010). Our AD cases did not show olfactory habituation. The POC is pathologically involved in the early AD stage (Wilson et al., 2007), the dysfunction of POC might appear as the increased olfactory threshold and lost olfactory habituation. These signs could potentially be used as markers for POC involvement and diagnosis of early AD. For the subjects who showed impaired olfactory function in MCI (Hagemeier et al., 2016), even in some NCs, despite their normal or minimal changes of cognition, they should be given high priority to follow-up, so that to give early intervention (Growdon et al., 2015).

To simulate the clinical settings, we summarized the POC activation patterns across different concentrations into five types: ascending-descending type A, gradually ascending type B, gradually descending type C, high plateau type D, and low plateau type E. Most (90.9%) AD patients showed the type B or E pattern of activation, which indicated an impaired olfactory function. More interestingly, 29/46 (63.04%) of the MCI, even 13/44 (29.55%) of the normal subjects also showed impaired habituation in the POC. This showed that the activation pattern of POC was a sensitive mark for evaluating olfactory function. Evidence has shown that some MCI subjects with olfactory dysfunction might be the early-stage AD patients (Petersen et al., 2001b; Gauthier et al., 2006; Jack et al., 2010). These MCI and NC subjects can be separated by the activation pattern of olfactory fMRI. We estimate that the MCI subjects with normal habituation in our study are not early AD and have normal POC function. Those MCI and NC with POC dysfunction might have POC pathological changes and follow-up is needed.

There were still some limitations of our study. First, we tried to use habituation effect to evaluate the olfactory function instead of the activation number and show better diagnostic efficiency on the AD subjects. But we did not have a gold standard for the olfactory function. The behavior smell tests were subjective methods which might also be different with the fMRI results. So we only assumed that the AD had an impaired olfactory function, whereas the normal controls had a normal olfactory function. The diagnostic efficiency was compared to distinguish the AD and NC groups. And the most diagnostic difference of these two methods should be obvious at the mild impaired olfactory function subjects. Such as, 29/46 (63.04%) of the MCI, even 13/44 (29.55%) of the normal subjects was found impaired habituation, follow-up of these subjects might can verify the benefits of the new method. Second, despite our results initially showing that the habituation effect could be helpful to evaluate the olfactory function, but its appearance characteristics at more different concentrations, such as a higher concentration should do further studies. This further research might reveal the mechanism of habituation. At last, we used a 15% activation difference to separate the different activation types. Actually, we had compared the different quantitative standard from 10, 15, and 20%. Only one normal subject and one AD patient had different activation types between 10% and 15% but no statistical difference (Supplement 2). All the activation types were same for the 15 and 20%. So, 15% might be sensitive to separate the different activation types for our data, but this might be different with different study conditions and labs. Other data analytic methods, such as the Support Vector Machine might be helpful to catch more activation information among these different concentrations.

## Conclusion

The olfactory fMRI with different odorant-concentration stimulants can be used to evaluate the POC function, and the activation patterns of POC might be a sensitive marker for detecting the POC pathological involvement and earlier stage AD.

## Ethics Statement

This study got approval of the research ethics committee of Tianjin First Central Hospital and all methods were performed in accordance with the relevant guidelines and regulations.

## Author Contributions

HZ, DJ, JY, ZW, YZ, and HN contributed to the examination of the subjects and data processing. JY, HZ, DJ, and YL contributed to the design and writing of the manuscript.

## Conflict of Interest Statement

The authors declare that the research was conducted in the absence of any commercial or financial relationships that could be construed as a potential conflict of interest.
