# Supplementary material for: Olfactory fMRI Activation Pattern Across Different Concentrations Changes in Alzheimer’s Disease
Source: Front Neurosci. 2019 Jul 30;13:786. doi: 10.3389/fnins.2019.00786 (PMC6682702; doi:10.3389/fnins.2019.00786)
Supplement: Supplementary file 1 [file Table_1.doc]

The Olfactory fMRI Activation Maps and Table of Normal Control, MCI and AD Groups

Supplement Figure 1 The Olfactory fMRI Activation Maps of Normal Control Group.


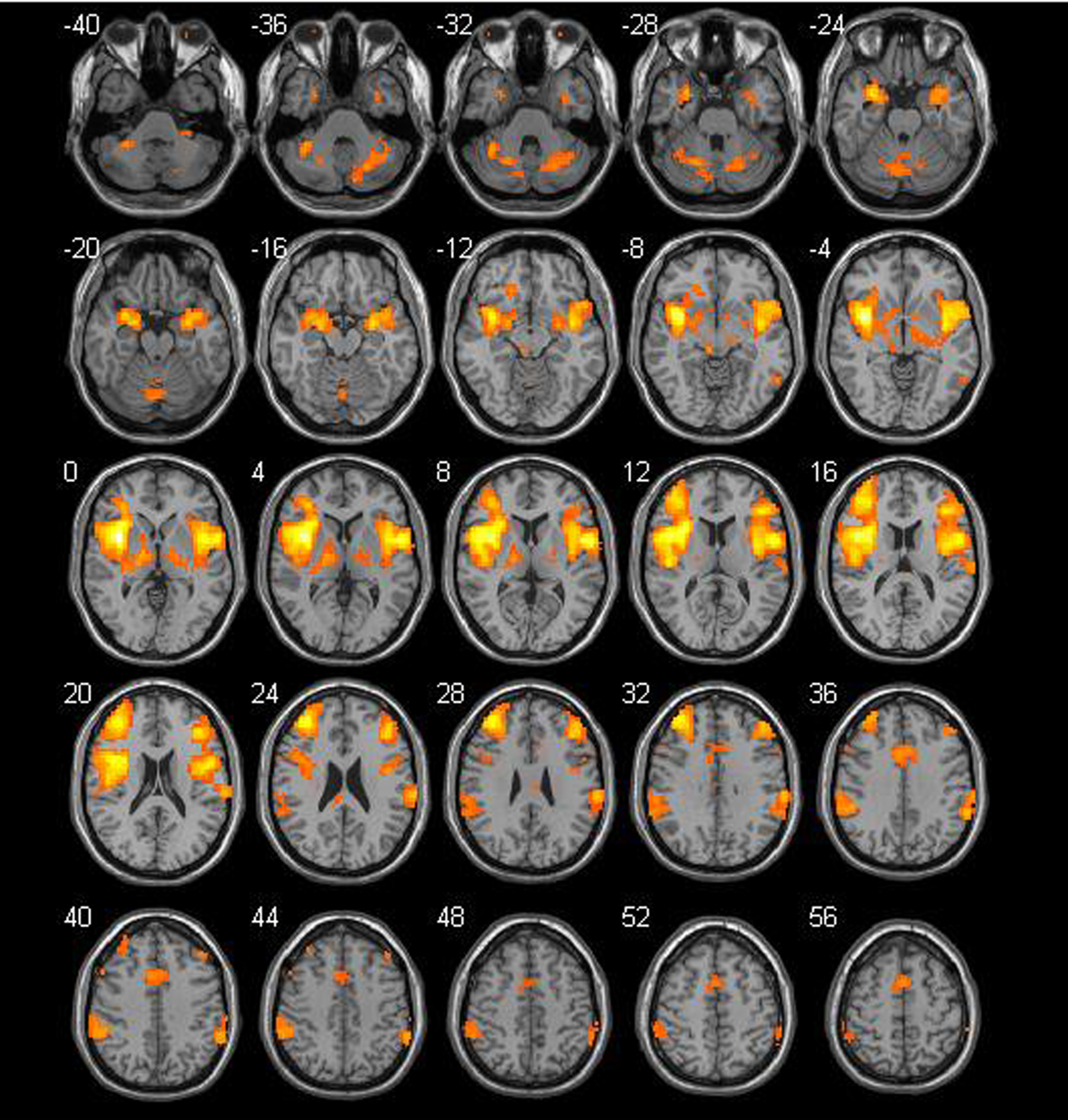


| Supplement Table1 The Olfactory fMRI Activation Clusters of Normal Control Group. | | | | | | |
| --- | --- | --- | --- | --- | --- | --- |
| **Location** | **MNI Coordinates** | | | **t-value** | **Activated voxels** |  |
|  | **x** | **y** | **z** |  |  |  |
| Right insula | 39 | 8 | 1 | 9.37 | 3292 |  |
|  | 45 | 8 | 13 | 7.49 |  |  |
|  | 35 | 8 | 16 | 7.23 |  |  |
| Left insula | -54 | 5 | 7 | 7.76 | 2341 |  |
|  | -42 | 5 | 1 | 6.97 |  |  |
|  | -39 | -4 | -5 | 6.22 |  |  |
| Right cerebellum | 39 | -46 | -35 | 4.73 | 257 |  |
|  | 21 | -54 | -29 | 4.52 |  |  |
|  | 33 | -46 | -41 | 4.39 |  |  |
| Superior Frontal Gyrus | 3 | 8 | 58 | 4.63 | 261 |  |
|  | 5 | 14 | 37 | 4.44 |  |  |
|  | -3 | 17 | 37 | 4.42 |  |  |
| Left cerebellum | -21 | -67 | -35 | 4.42 | 196 |  |
|  | -15 | -57 | -29 | 4.26 |  |  |
|  | -35 | -58 | -32 | 4.24 |  |  |
| Left Inferior Temporal Gyrus | -57 | -58 | -8 | 4.12 | 13 |  |
| Left Medial Globus Pallidus | -12 | -2 | -5 | 3.91 | 7 |  |
| Right Posterior Cingulate | 6 | -31 | 25 | 3.89 | 10 |  |
| Right Cingulate Gyrus | 12 | -37 | 25 | 3.59 |  |  |

Supplement Figure2 The Olfactory fMRI Activation Maps of MCI Group.


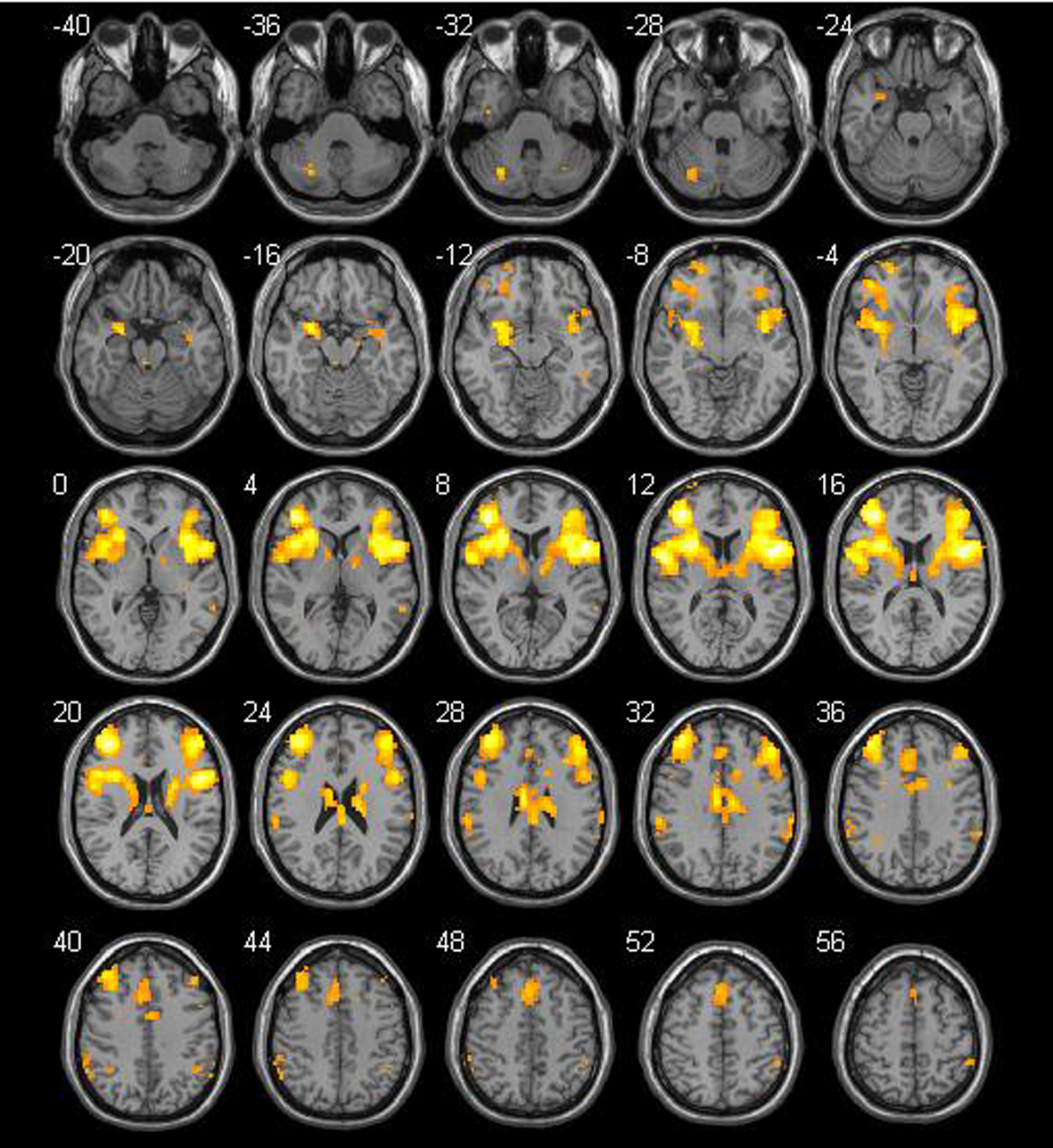


| Supplement Table2 The Olfactory fMRI Activation Clusters of MCI Group. | | | | | |
| --- | --- | --- | --- | --- | --- |
| **Area** | **MNI Coordinates** | | | **t-value** | **Activated voxels** |
|  | **x** | **y** | **z** |  |  |
| Left Frontal Lobe of Sub-Gyral | -45 | 5 | 16 | 7.36 | 4998 |
|  | -51 | 11 | 7 | 7.16 |  |
| Right Middle Frontal Gyrus | 21 | 56 | -5 | 4.73 | 38 |
| Right SupraMarginal | 63 | -31 | 28 | 4.45 | 94 |
|  | 63 | -43 | 40 | 3.71 |  |
|  | 60 | -40 | 49 | 3.53 |  |
| Right Cerebellum Posterior Lobe | 30 | -67 | -32 | 4.40 | 32 |
| Left SupraMarginal | -63 | -31 | 31 | 4.20 | 45 |
|  | -60 | -46 | 31 | 4.06 |  |
| Left Superior Temporal Gyrus | -60 | -46 | 4 | 4.00 | 9 |
| Right Middle Frontal Gyrus | 30 | 68 | 10 | 3.98 | 8 |
| Left Insula | -39 | -22 | -2 | 3.83 | 7 |
| Right Frontal Lobe of Sub-Gyral | 30 | 5 | 37 | 3.80 | 4 |
| Left Middle Frontal Gyrus | -33 | 2 | 37 | 3.79 | 5 |
| Midbrain | 3 | -31 | -20 | 3.77 | 5 |
| Left Parietal Lobe | -51 | -43 | 55 | 3.74 | 23 |
| Left Inferior Parietal Lobule | -45 | -49 | 40 | 3.53 |  |

Supplement Figure3 The Olfactory fMRI Activation Maps of AD Group.


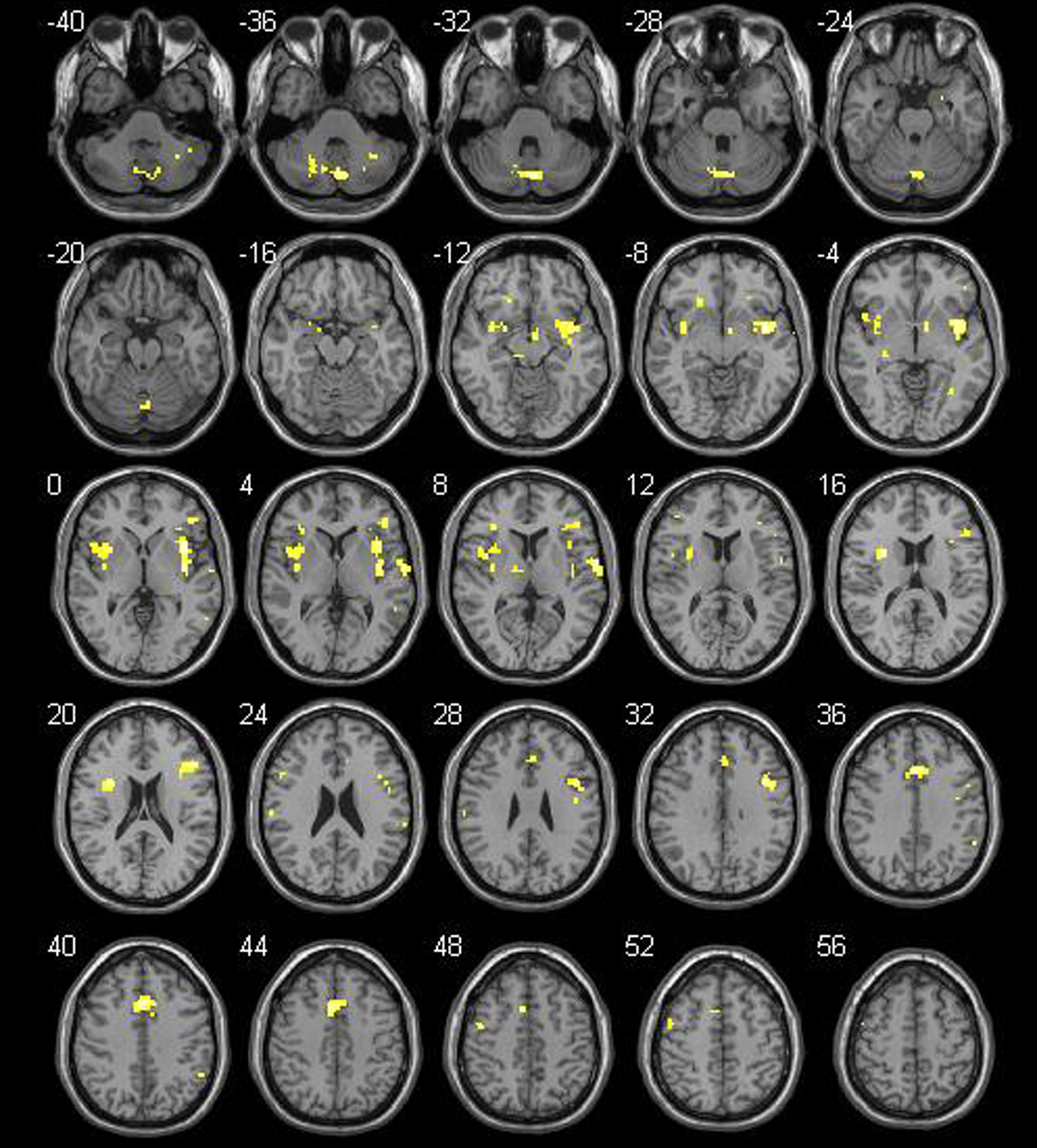


| Supplement Table3 The Olfactory fMRI Activation Clusters of AD Group. | | | | | |
| --- | --- | --- | --- | --- | --- |
| **Area** | **MNI Coordinates** | | | **t-value** | **Activated voxels** |
|  | **x** | **y** | **z** |  |  |
| Left Insula | -39 | -1 | -86 | 4.90 | 181 |
|  | -35 | 11 | 1 | 4.80 |  |
|  | -35 | -7 | 1 | 4.54 |  |
| Cerebellum Posterior Lobe | 0 | -70 | -35 | 4.80 | 124 |
|  | -3 | -70 | -26 | 4.33 |  |
|  | 9 | -70 | -29 | 4.11 |  |
| Right Cingulate Gyrus | 3 | 17 | 40 | 4.63 | 104 |
|  | 9 | 11 | 46 | 4.37 |  |
|  | 0 | 29 | 28 | 3.91 |  |
| Left Superior Temporal Gyrus | -63 | -7 | 7 | 4.45 | 46 |
| Right Frontal Lobe of Sub-Gyral | 33 | 8 | 19 | 4.37 | 172 |
| Right Insula | 35 | 11 | -2 | 4.28 |  |
| Right Precentral Gyrus | 42 | 5 | 7 | 3.95 |  |
| Left Inferior Frontal Gyrus | -45 | 23 | 19 | 4.16 | 38 |
| Left Frontal Lobe of Sub-Gyral | -33 | 20 | 19 | 3.87 |  |
| Left Middle Frontal Gyrus | -45 | 38 | -2 | 4.08 | 37 |
