# Supplementary material for: Olfactory fMRI Activation Pattern Across Different Concentrations Changes in Alzheimer’s Disease
Source: Front Neurosci. 2019 Jul 30;13:786. doi: 10.3389/fnins.2019.00786 (PMC6682702; doi:10.3389/fnins.2019.00786)
Supplement: Supplementary file 2 [file Table_2.doc]

Supplement Table4 The Different Quantitive Standard of Activation Pattern across Concentrations

| Groups |  | Type A | Type B | Type C | Type D | Type E | χ^2^ | *P* |
| --- | --- | --- | --- | --- | --- | --- | --- | --- |
| NC | 10% | 15 | 3 | 14 | 2 | 10 | 0.300 | 1.000 |
|  | 15% | 15 | 3 | 13 | 3 | 10 |  |  |
|  | 20% | 15 | 3 | 13 | 3 | 10 |  |  |
| MCI | 10% | 8 | 15 | 9 | 0 | 14 | 0.000 | 1.000 |
|  | 15% | 8 | 15 | 9 | 0 | 14 |  |  |
|  | 20% | 8 | 15 | 9 | 0 | 14 |  |  |
| AD | 10% | 3 | 16 | 2 | 0 | 23 | 0.314 | 0.999 |
|  | 15% | 2 | 16 | 2 | 0 | 24 |  |  |
|  | 20% | 2 | 16 | 2 | 0 | 24 |  |  |
